# Modeling Au Nanostar Geometry in Bulk Solutions

**DOI:** 10.1021/acs.jpcc.2c07520

**Published:** 2023-01-12

**Authors:** William Morton, Caoimhe Joyce, Jonny Taylor, Mary Ryan, Stefano Angioletti-Uberti, Fang Xie

**Affiliations:** Department of Materials, Imperial College London, LondonSW7 2AZ, U.K.

## Abstract

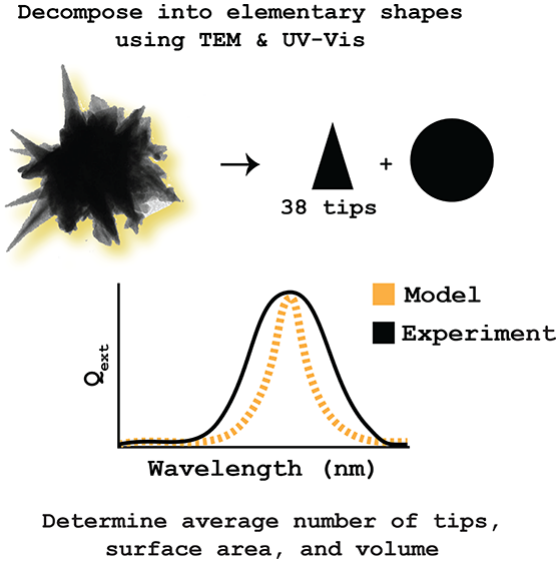

The findings within make it possible to reference gold nanostars based on their geometric properties, similar to how a radius describes a nanosphere, rather than just the LSPR of the structure—the current practice. The average tip approximation presented reduces the complexity of nanostars in discrete dipole approximation simulations. By matching the projected area and LSPR of the modeled nanostars to synthesized nanostars, the volume, surface area, and number of tips can be approximated without a lengthy characterization process. Knowing the nanoparticle geometry can determine drug carrier capacity, an approximate number of hot spots for EM imaging, and how the particle will interact with cells. The geometric data obtained will drive the biological application and increase the usability of this particle class.

## 1 Introduction

Electron microscopy and UV–vis spectroscopy have been crucial for developing correlations between the optical and geometric properties of nanoparticles.^1^ Shape factors have been exploited to relate the aspect ratios of nanorods to localized surface plasmon resonances (LSPRs), determine the transmissivity of nanohole arrays, and relate the aspect ratio of gold nanostar tips to LSPRs.^2−5^ To verify the existence of these relationships, electromagnetic modeling is typically performed using an approximated solution to Maxwell’s equations for the particle.^6,7^ When used in unison, these three techniques can predict the optical properties of nanoparticles and determine their ideal application.

**Figure 1:**
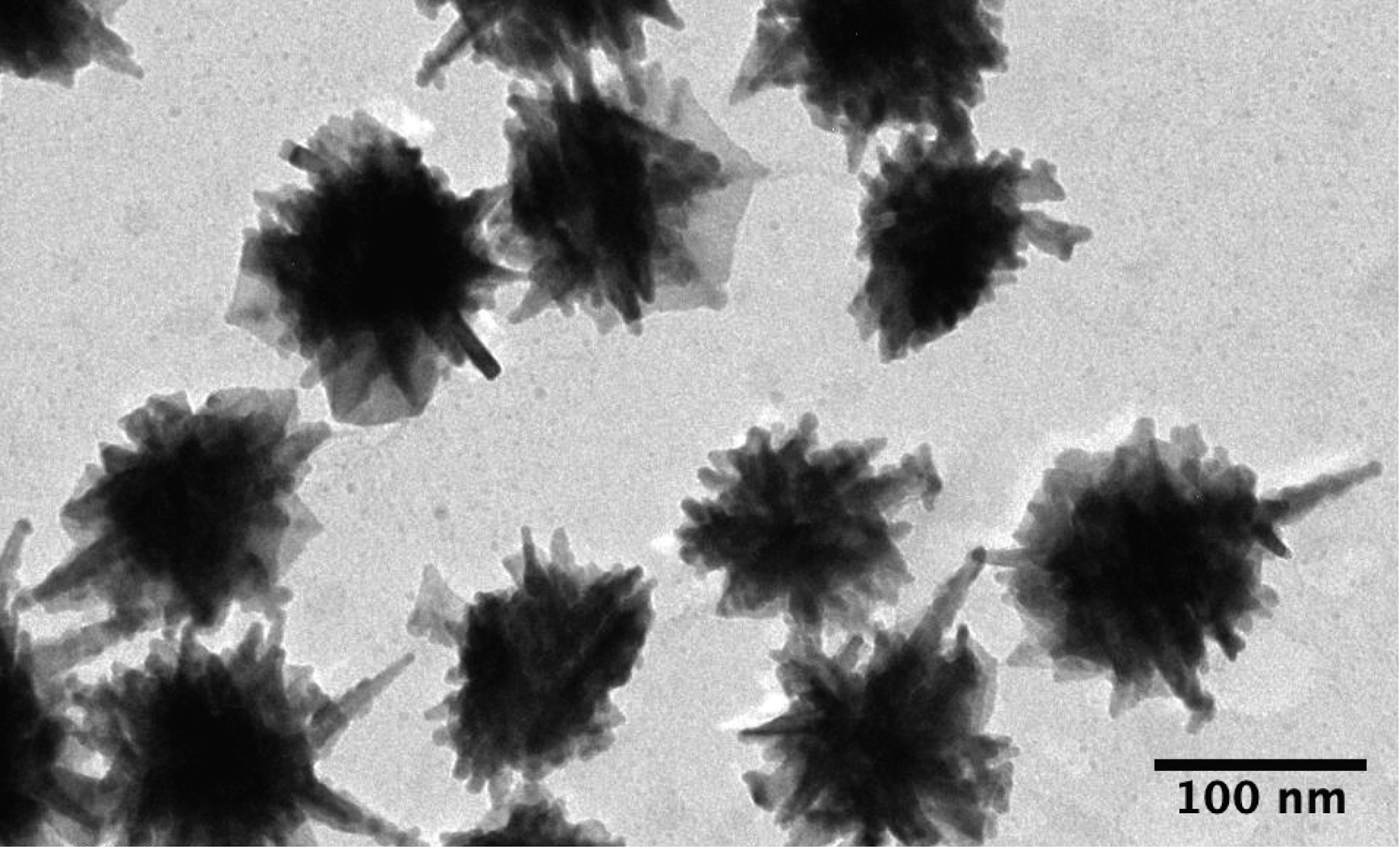
BF-TEM image of a solution of AuNS with a polydispersity of 0.036, as measured by dynamic light scattering. Despite the monodispersity of the solution, a range of core radii and tip lengths can be identified.

Plasmonic nanoparticles with one or two geometric factors, like nanospheres, nanorods, and nanoshells, are easily described in bulk. Consider a gold nanosphere solution with an average radius of 15 nm. These require only one model to verify the bulk solution’s optical properties, and the absorption spectra’s breadth would be attributable to the polydispersity of the radii. AuNS (AuNS) suffer from geometric factors of two different shapes, a spherical core and conical tips, along with a volumetric ratio between the two. Additionally, acquiring the geometry of the tip in a monodisperse solution requires averaging the geometric properties of the tips on one star and then averaging that over many stars (Figure 1). Because of the complexity of this process, no electromagnetic model currently exists to describe a bulk solution of AuNS geometrically; they are only described by their resonance wavelength.

Angle-resolved TEM images have given the most accurate depictions of individual AuNS, at the cost of a significant amount of experimental time.^8−11^ Through these studies, it has been shown that the resonance modes of a complex structure are based on hybridization between the resonance mode(s), or eigenstates, of the elementary parts that make up that structure.^12^ In the case of a gold nanostar, these elementary parts are the core (sphere) and tips (cones).^13^ While the structures of individual nanostars can be explored and modeled, the results are not necessarily representative of the bulk solution.^14^

In academia, AuNS are predominantly used either as photothermal agents due to their ability to absorb light and generate heat at longer wavelengths or for their localization of electric fields at their tips. The latter is helpful for applications such as surface-enhanced Raman scattering and fluorescence-based sensing.^14,15^ A large number of tips not only can provide a high density of electric field enhancement points but also can introduce steric hindrance, leading to fewer bound molecules. Therefore, a method of determining the optical properties, and the approximate number of tips, on a gold nanostar is desirable.

Beyond photothermal therapy, fluorescence, and Raman sensing, AuNS have newfound uses as drug delivery agents, as their geometry can enhance their ability to cross biological barriers.^16,17^ Quantifying the approximate number of tips can give insight into the particle’s surface area, a crucial step for understanding their utility as drug carriers. However, depending on the tip’s geometry, there can be an increase in uptake into cells, degradation of cell membranes, or higher adsorption to the membrane.^16^ It is increasingly necessary to correlate the geometric characteristics of these nanostructures not only to their optical properties but also to their cellular uptake, immune system evasion, and cargo delivery.^17,18^

Although there is great interest in finding a use for AuNS, their commercial implementation has been limited, in large part due to their poor reproducibility and complex characterization.^19^ A characterization mechanism is needed to determine the properties of a bulk solution of nanostars to better realize their use. The purpose of this study is to reduce the complexity of modeled AuNS to determine their bulk optical and geometric properties. A simple method is presented to generate average geometric and optical information about nanostars based on UV–vis spectra and TEM images, setting a precedent to include electromagnetic models as a necessary characterization method to complement experimental observables of nanostars.

## 2 Methods

Gold nanostars are constructed by generating a point-based model on a cubic lattice for discrete dipole approximations (DDA) using the core radius, tip length, tip radius, and the number of tips. Points are treated as dipoles, and the polarization, *P*, of each dipole at point *j* is found with respect to every dipole within the system. Once every polarization has been calculated, the extinction, absorption, and scattering efficiencies (*Q*_ext_, *Q*_abs_, and *Q*_sca_) can be found using the following equations:^20^
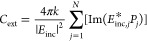
1
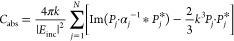
2
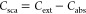
3
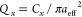
4In the preceding equations, α is the polarizability of the material, *k* is the wavenumber of interest, *k* = 2π/λ where λ is the wavelength of light in vacuo, *E*_inc_ is the incident electric field, *E*_inc,*j*_ is the incident electric field at point *j*, and *a*_eff_ is the effective radius of the shape. In eq 4, the subscript “*x*” is replaced with “ext”, “sca”, or “abs”, as all three properties are found by dividing the cross section, *C*_*x*_, by the effective area of a circle with radius = *a*_eff_, to obtain the efficiency *Q*_*x*_.

In the nanostar model, the core is a sphere, and the tips are conical protrusions, with the length and radius measured at the point of intersection tangent to the core. Each tip is added at a random orientation using a Euler rotational matrix. The edges of the cone are then extended to intersect with the core. Modeled gold nanostars vary in size but are always composed of more than 10^4^ dipoles to meet the interdipole spacing criterion, |*m*|*kd* < 0.5. In all calculations, the largest value of |*m*|*kd* was 0.0384.

Gold nanostars are run in DDSCAT, a free DDA simulation software, surrounded by an aqueous environment with refractive index (RI) = 1.33.^21^ The dispersion information for Au is the commonly used Johnson and Christy dielectric constants.^22^ Each particle is generated 30 times and exposed to just one polarization. Because tips are placed randomly around the particle, this ensures that an average, “bulk” response is considered. In all the following sections, the gold nanostars will be described in two elementary parts: the core and the tips.

To experimentally create the gold nanostars used for the study, gold chloride trihydrate (HAuCl_4_·3H_2_O), silver nitrate (AgNO_3_), sodium citrate tribasic dehydrate, and l-ascorbic acid (AA) was purchased from Sigma-Aldrich, United Kingdom. Hydrochloric acid (37%) was acquired from VWR International, United Kingdom. The heterobifunctional polymer thiol-PEG(7500)-amine was purchased from JenKem Technology, USA. For all synthesis and experiments, Milli-Q Type 1 ultrapure water was used.

Gold nanostars were synthesized via a seed-mediated method. To make the initial seeds from which the nanostars were grown, 100 mL of a 0.25 mM aqueous HAuCl_4_·3H_2_O solution was heated to boiling in a 250 mL Erlenmeyer flask under magnetic stirring. Then, to make Au seeds of 41 nm, 0.25 mL of a 3.3% (w/v) aqueous sodium citrate solution was added to the flask under vigorous stirring. After approximately 2 min, the solution appeared blue-purple, which quickly turned a deep pink-red color. The solution was left to stir under heat for a further 10 min to ensure full completion of the seed synthesis. This solution was then cooled in an ice bath, and its volume was made back up to 100 mL with Milli-Q water. The seeds were stable and could be stored long term at 4 °C. Two sets of Gold nanostar were grown using the seeds and growth solutions of different Au concentrations. Growth solutions were added by first mixing 10 mL of a 0.2 mM (“small”), or a 0.6 mM (“large”), HAuCl_4_·3H_2_O solution containing 20 μL of 1 M HCl and 600 μL of seeds. While this solution was vortexed at high speed, 60 μL of 10 mM AgNO_3_ followed quickly by 100 μL of 100 mM AA. After 30 s of vortexing, the fully formed nanostars were then stabilized with 100 μL of a 10 mM PEG spacer (7500 Da) containing a thiol group at one end and an amine at the other.

The absorption spectra of the seeds and nanostars were recorded using a Cary 5000 UV–vis–NIR spectrophotometer in the wavelength range between 400 and 1200 nm. For approximate determination of nanostar hydrodynamic radius (Rh) and uniformity, dynamic light scattering was performed using a Malvern Zetasizer Nano ZSP. The structure and morphology of the nanostar samples were determined using a JEOL JEM2100 Plus transmission electron microscope operating at an acceleration voltage of 200 kV. Size measurements were collected using ImageJ. Gold concentrations were determined by inductively coupled plasma optical emission spectroscopy (ICP-OES) using a Thermo Scientific iCAP 6000 Series ICP spectrometer. Gold nanostars were centrifuged, filtered, and then dissolved by adding 1 mL of concentrated aqua regia (prepared freshly from HCl and HNO_3_ in a 3:1 molar ratio) to 1 mL of gold nanostar suspensions. After 10 min, 6 mL of Type 1 Ultrapure water was added, making the final volume 8 mL before measurement.

## 3 Results and Discussion

### 3.1 Nonuniform Tips

To derive a model that can predict the shape of gold nanostars, it is crucial to know what level of detail the model requires. First, the influence of nonuniform tips on the extinction spectra of a gold nanostar. A core radius of 24 nm is chosen to represent a realistically synthesizable structure without taking too much computational time due to size. Tip length and radius are randomly chosen from a Gaussian distribution, centered on an average tip of length = 24 nm and radius = 12 nm, with standard deviation increasing from 1 to 30% of the desired length or radius.

**Figure 2:**
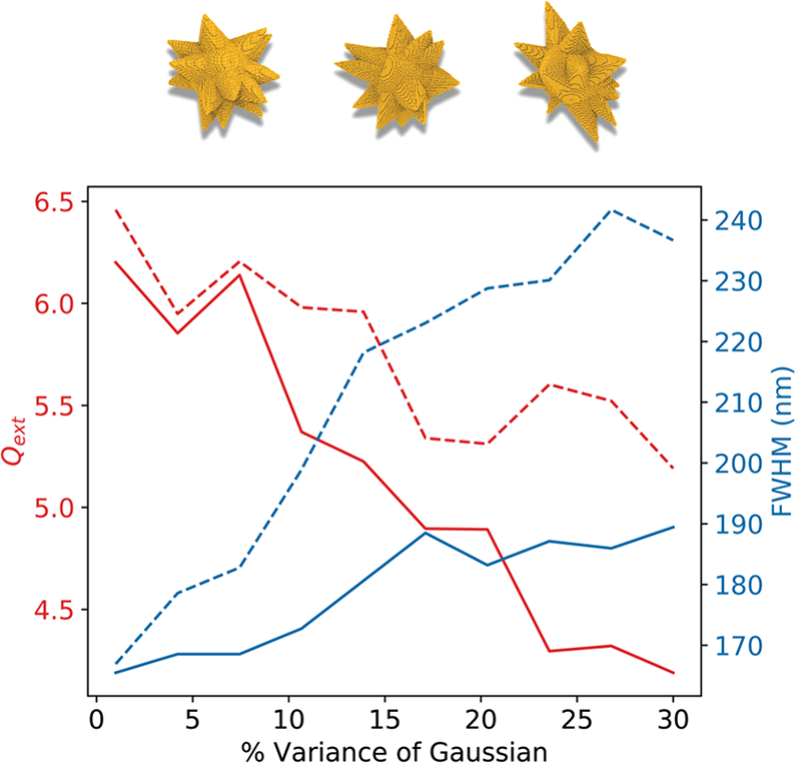
Change in *Q*_ext_ (red) and full width at half-maximum (blue) with increasing nonuniformity of tip radii (dashed) and length (solid). Shown above are example DDA models of nanostars with increasing tip variability.

A substantial decrease in *Q*_ext_ and a broadening of the full width at half-maximum occurs as the variance of the Gaussian increases, and particles lose uniformity (Figure 2). Competing LSPRs from nonuniform tips are suspected to be responsible for these effects, which has been shown to be true for nonuniform particle solutions in the literature.^23^ The LSPR and volume change as nonuniformity increases, but these shifts are small and without correlation, with the range being only 17 nm for the LSPR and 7.2 × 10^–3^ nm^3^ for the volume.

Because nonuniformity in the width, length, or both results in an increase in the full width at half-maximum, it is impossible to discern from which geometric factor the experimental full width at half-maximum originates. In bulk solutions, variance can also be expected in core size, widening the breadth of the extinction spectra. Therefore, when comparing the modeled data to the experimental UV–vis spectra, it is unnecessary to include nonuniform tips; an average tip will suffice.

### 3.2 Number of Tips

**Figure 3:**
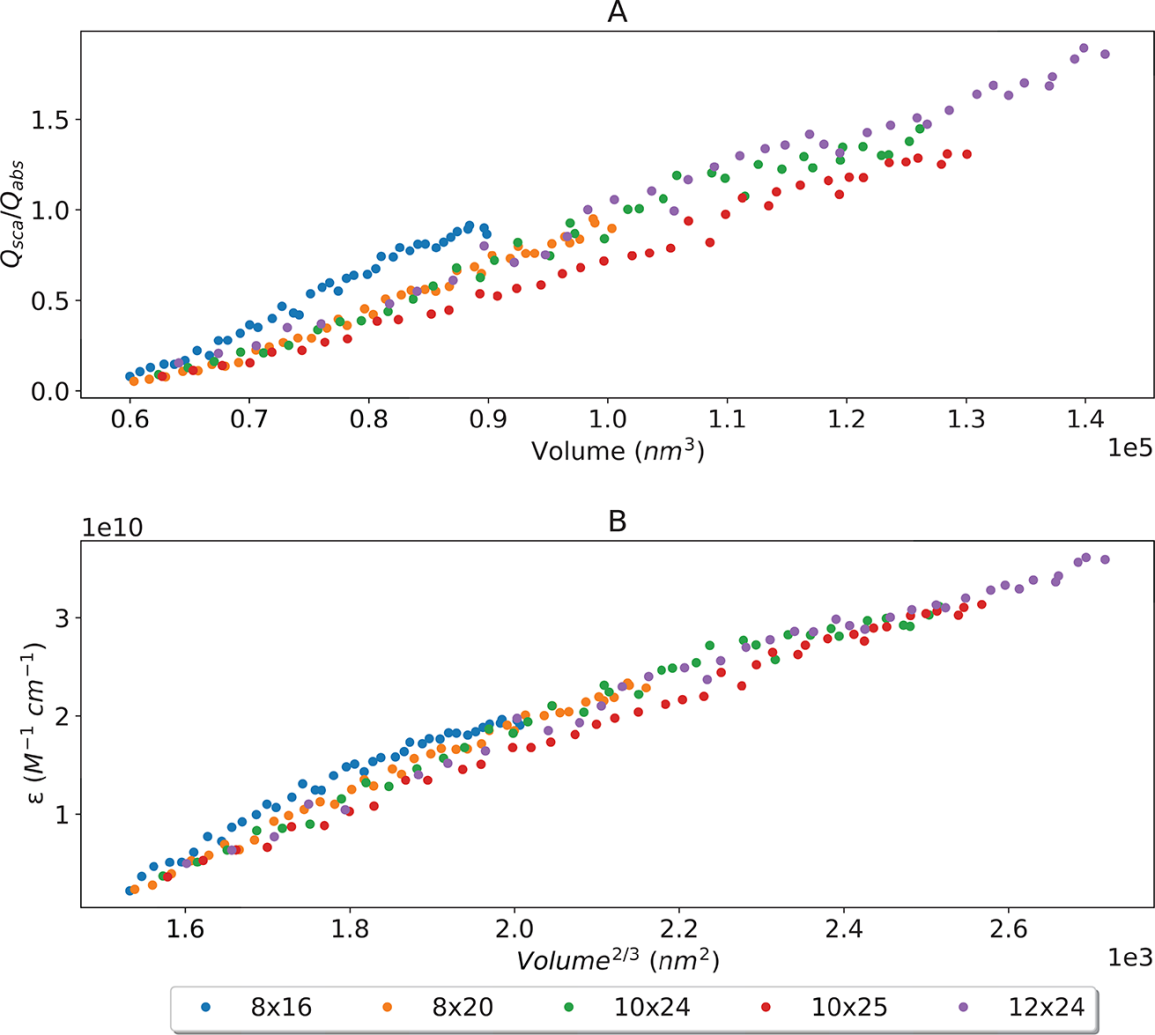
Nanostar with core radius of 24 nm and various tip geometries (shown in legend as radius × length). As more tips are added, the overall volume increases, showing (A) a linear relationship between molar absorptivity and volume^2/3^ and (B) a linear increase in the dominance of *Q*_sca_ on the extinction spectra.

Increasing the number of tips on a core of radius = 24 nm, from 1 to 40, causes an increase in the *Q*_ext_ at the LSPR (Figure S2). At lower volumes, *Q*_ext_ is dominated by *Q*_abs_, but as the particle size increases, the scattering contribution increases, similar to gold nanospheres (Figure 3A).^24,25^ Regardless of the tip dimensions or aspect ratio, the same patterns are followed but with different rates of increase depending on the tip used. The slope is likely related to the difference in *Q*_ext_ at the resonance of the tips themselves, which is highest for the tip radius and length of 10 and 25 nm, respectively, and lowest for the tip with radius and length of 8 and 16 nm, respectively (Figure S1). A useful takeaway from the single graph is the ability to compare the scattering potential between two solutions of nanostars based on minimal calculations.

In comparison, the molar extinction increases linearly with volume, regardless of tip dimensions, using the empirical relationship suggested experimentally by De Puig et al. and shown in Figure 3B.^8^

### 3.3 Core Size

**Figure 4:**
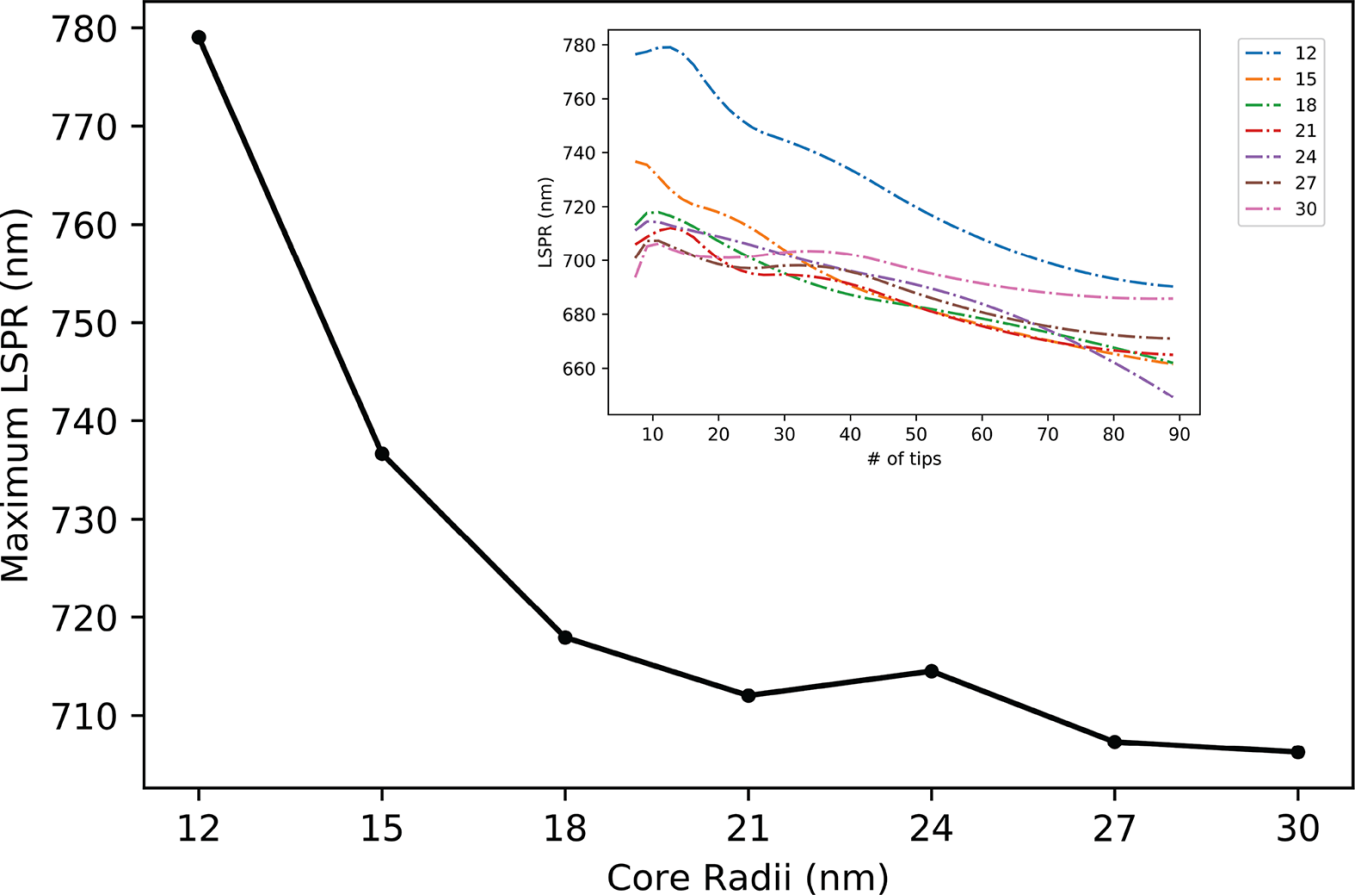
Change in LSPR for varying core radii with 5 tips. All tips have identical dimensions. The inset shows how LSPR varies with an increase in tips for spheres with different radii.

The number of tips changes the strength of the response at the resonance while also strongly impacting the wavelength of the resonance. By varying the core size and adding identical tips, the LSPR of the structure shifts toward wavelengths of higher energy: toward the resonance of the unhybridized elementary structures. As seen in Figure 4, particles with larger cores shift less, which could be associated with a large number of electrons present in the core. Hybridization strength depends on the contribution of electrons from each elementary shape. A large core particle remains excited by wavelengths of higher energy, with the smallest range of LSPRs out of the radii considered. A robust model for a bulk solution of AuNS must consider the volumetric ratio between the core and tips.

The core radius of the nanostar will limit the accuracy of all nanostar models. The dynamic range should be considered the difference between the largest resonance wavelength for a given tip geometry on a core, typically 5 tips, and the saturation resonance as many tips are added. In the systems studied above, a core radius of 12 nm has a dynamic range of 88 nm, while a core radius of 30 nm only has a dynamic range of 20 nm.

Figure 4 also shows that more than obtaining the elementary resonances of the structures alone is needed to predict the optical properties of a hybridized structure. The volumetric ratio between the core and tips changes the strength of the optical response and the resonance itself. Unfortunately, when analyzing TEM images, the number of tips on a nanostar is often difficult to discern. To date, the best approximation for the volume of a nanostar is obtained by using the volume of a sphere with the same projected area as the nanostar, the effective radius, *a*_eff_. A comprehensive model to determine exact geometric features should be based on the observable *a*_eff_ but should also be validated by modeling optical properties.

### 3.4 Application

**Figure 5:**
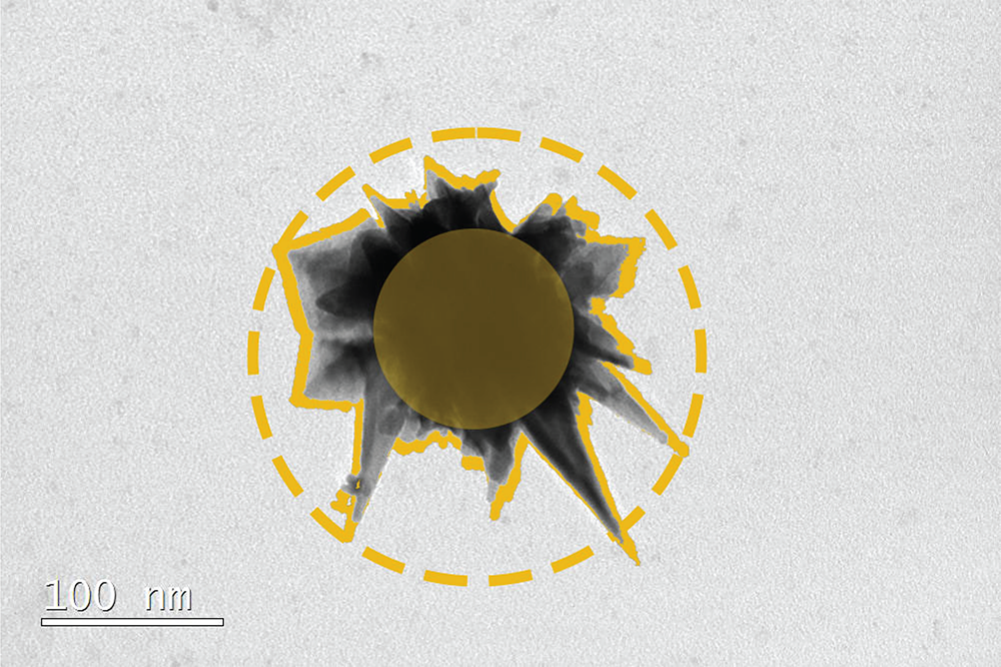
TEM image of gold nanostar from the large growth solution, depicting the core and maximum enclosed sphere for calculating the maximum tip length. Additionally, the cross-sectional area is seen around the star to calculate *a*_eff_.

A workflow was developed using the limitations and findings above, predicting the average geometry of gold nanostars in two bulk solutions grown using “small” and “large” gold seeds. The projected area of 20 gold nanostars per solution is measured using TEM images. The core radius is found by creating the largest perfect circle within the star’s core. The maximum radius is that of the circle that passes through the three largest tips. An example of how the geometric measurements are gathered can be seen in Figure 5. Experimental observables, such as the hydrodynamic radius and concentration of Au in the solution, along with the measured geometric properties used in the next step can be seen in Table 1.

**Table 1:** Experimentally Observed Properties of Each Nanostar Solution

solution	*R*_h_ (nm)	conc. (ppm)	core radius (nm)	max radius (nm)	projection area (nm^2^)
small	92	0.29	39	83	10.6 × 10^3^
large	111	0.60	52	113	18.6 × 10^3^

Nanostars are modeled within the measured parameters, with tips at random orientations, and the average projection area is used as a metric of success. Nanostars with similar projection areas are then modeled in DDSCAT to determine their LSPR.

**Figure 6:**
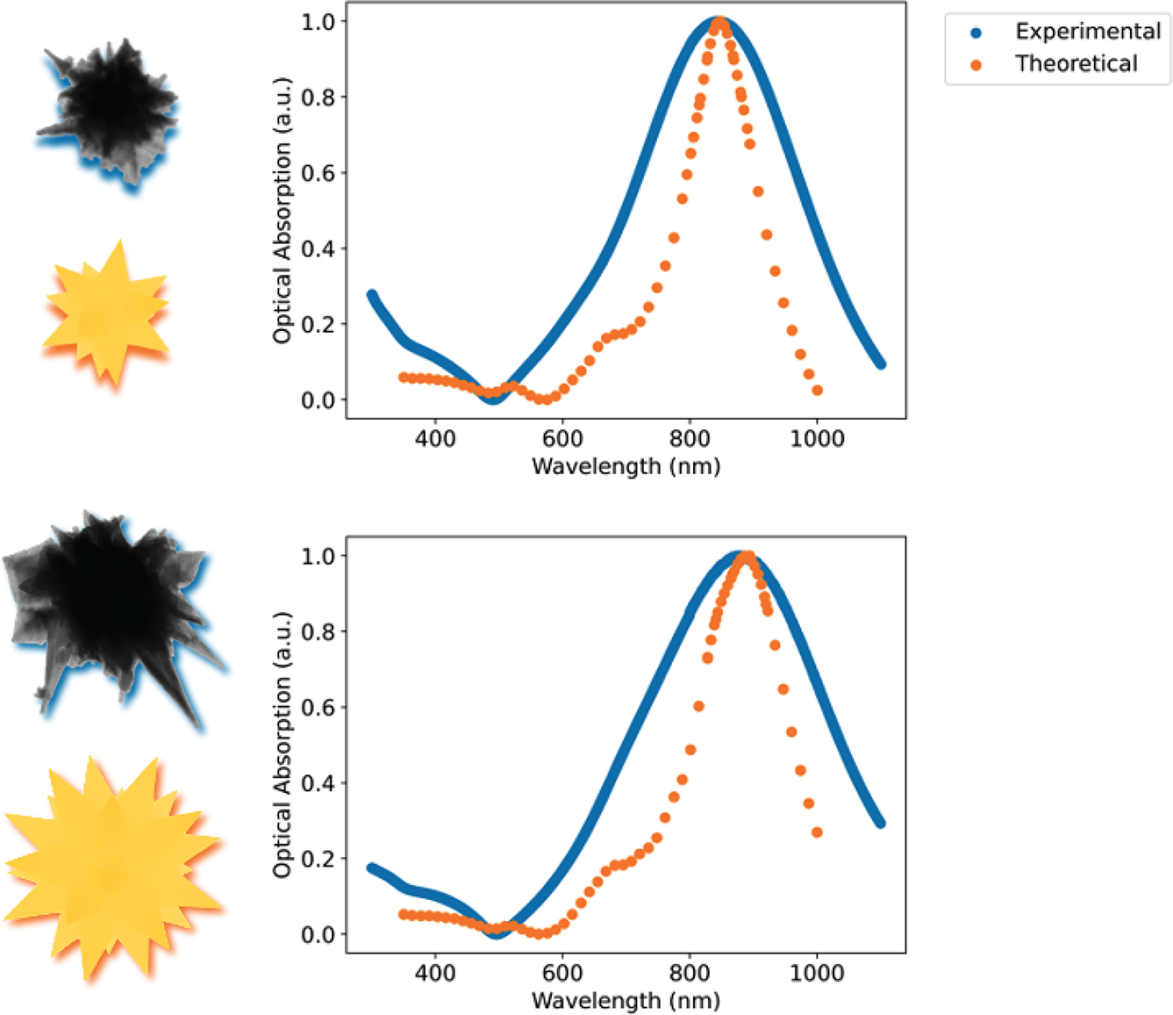
UV–vis absorption spectra of the small (top) and large (bottom) nanostars, in comparison to the theoretical spectra of the modeled nanostars referenced in Table 2.

Of the nanostars modeled, only one nanostar for each solution could reproduce the experimental resonance wavelength, the geometry of which is seen in Table 2. However, should multiple nanostars have the same resonance and projected area, it would be up to the researcher to determine how best to treat the data (e.g., by averaging the properties of the nanostars or eliminating unreasonable possibilities). Figure 6 shows the extinction spectra of the modeled nanostars compared to those gathered from the experiment. The resonance wavelengths are in good agreement, suggesting that a representative average tip size, and the ratio between core volume and tip volume, were found. However, the full width at half-maximum of the synthesized AuNS solution is much larger than the modeled nanostar. Variance in core and tip dimensions throughout the solution results in the discrepancy. As with most solutions of nanoparticles, the full width at half-maximum of the extinction spectra can be a good measure of particle uniformity and should be taken into account when determining the application of the particle solution.

**Table 2:** Geometric Properties of the Modeled Nanostars Found to Be Representative of the Experimental Solution^a^

solution	ΔLSPR (nm)	tip length × radius (nm)	volume (nm^3^)	surface area (nm^2^)
small	3	39 × 14	4.23 × 10^5^	6.5 × 10^4^
large	8	62 × 26	10.94 × 10^5^	10.7 × 10^4^

a Change in LSPR between experimental and theoretical results is used as a metric for success.

The results above estimate that the volume of the successful nanostar candidate is 50% of that which would be expected from just measuring *a*_eff_. Nanostars with the same projection area were also found to have a 22% variation in volume, further suggesting that the use of *a*_eff_ alone is insufficient. Three times more growth solution was added to the “large” solution, resulting in particles that have an estimated 2.6× increase in volume over the “small” growth solution. The authors hypothesize that unreacted gold may be responsible for the discrepancy.

## 4 Conclusion

Introducing a reliable characterization method that complements experimental observables will lower the barriers to implementing this increasingly valuable particle class. The technique presented above outperforms typical methods of estimating the size of nanostars using purely experimental techniques. Specifically, the geometric properties, including particle volume, surface area, and the number of tips in a synthesized solution, can be predicted using UV–vis, TEM images, and the DDA method outlined above. By taking advantage of the unique optical properties of metallic nanoparticles, these complex structures can be characterized more completely.

## Data Availability

Raw data and code developed to generate nanostars can be found at: https://github.com/shakespearemorton/starscat.

## Supporting Information Available

Additional plots of optical properties (PDF)
