# Supplementary material for: Modeling Au Nanostar Geometry in Bulk Solutions
Source: J Phys Chem C Nanomater Interfaces. 2023 Jan 12;127(3):1680–6. doi: 10.1021/acs.jpcc.2c07520 (PMC9884095; doi:10.1021/acs.jpcc.2c07520)
Supplement: Supplementary file 1 — jp2c07520_si_001.pdf [file jp2c07520_si_001.pdf]

Modelling Au Nanostar Geometry in Bulk Solutions

William Morton,* Caoimhe Joyce, Jonny Taylor, Mary Ryan, Stefano Angioletti-Uberti, and Fang Xie

Department of Materials, Imperial College London, London, U.K.

E-mail: wm816@ic.ac.uk

1 Supplementary Figures

The main text breaks nanostars into two elementary shapes: spherical cores and cone-like tips. To fully understand the effects of hybridisation between these two structures, it is crucial to know how they interact with light individually. Figure S1 shows Q_{ext} for the cones used throughout the text. Each cone is exposed to two polarisations to capture the effect of the LSPR along the two main axes of the cone. The conditions used are the same as those in the main text, RI = 1.33 and Au is from the Johnson and Christy dataset.

Figure S2 shows the decomposition of Figure 3A into the measured Q_{abs} and Q_{sca}.

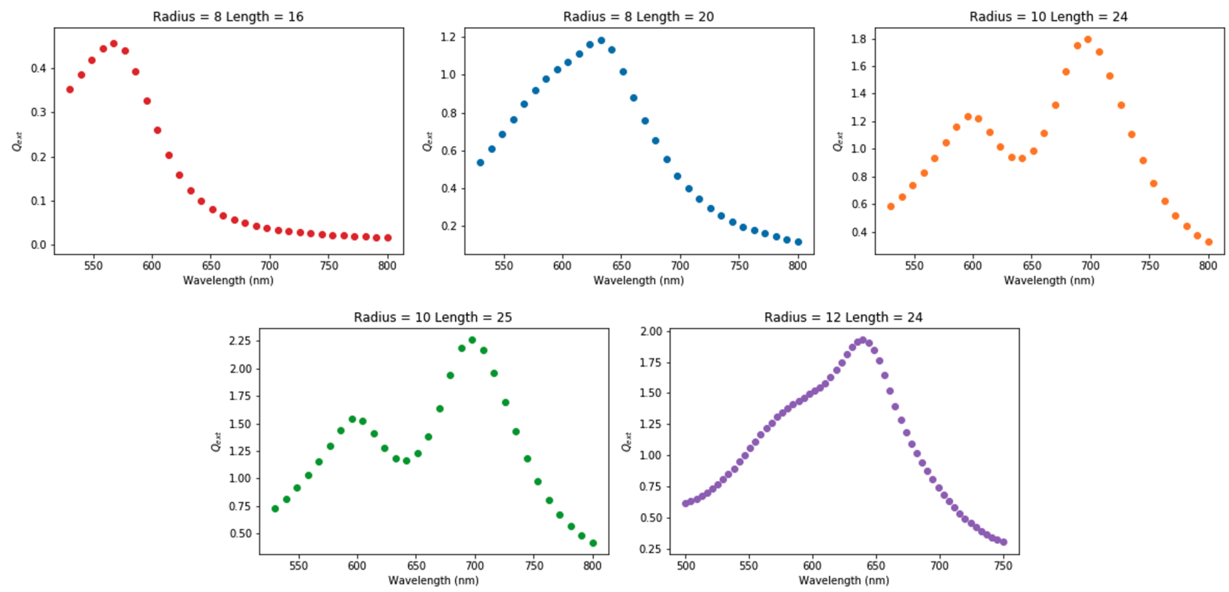

Figure S1: Q_{ext} for cones with various radii and lengths. These cones were placed on the cores mentioned in the main document.

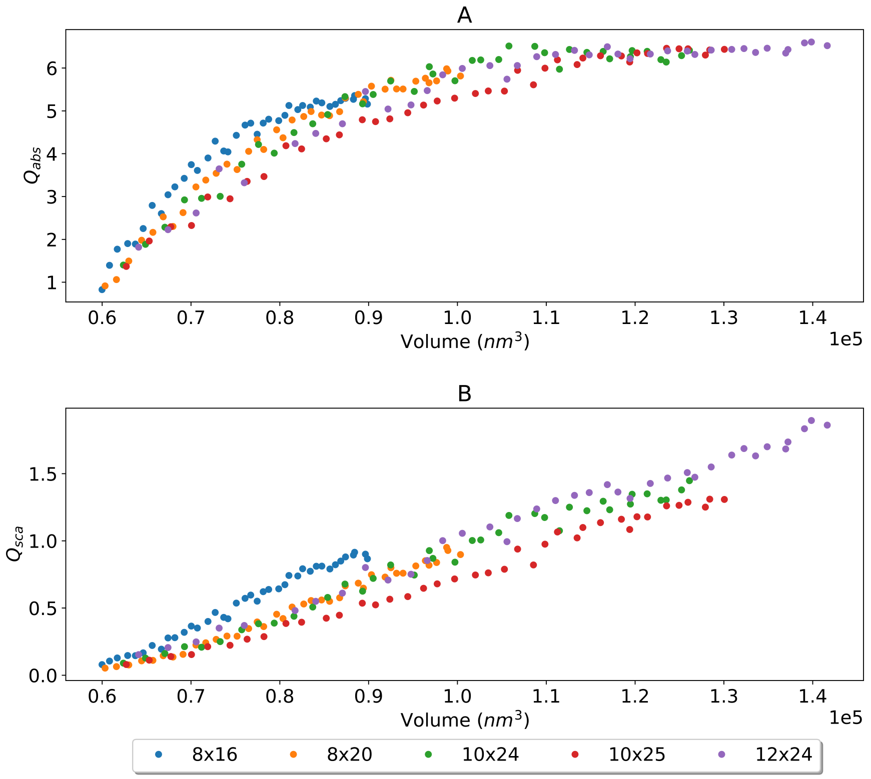

Figure S2: Q_{abs} and Q_{sca} for the system discussed in Figure 3. As Q_{ext} is the sum of the two properties above, this increases as well with volume.
